# Transmogrifying Anatomy Learning by Kaizen Strategies and Game-Based Education

**DOI:** 10.7759/cureus.64073

**Published:** 2024-07-08

**Authors:** Geetha S G, Rohini Motwani, Mrudula Chandrupatla, Punnapa Raviteja, Ariyanachi K

**Affiliations:** 1 Department of Anatomy, All India Institute of Medical Sciences, Bibinagar, Hyderabad, IND

**Keywords:** game-based learning, continuous improvement, interactive learning, kaizen, anatomy

## Abstract

Introduction: The study explores the significance of continuous improvement through Kaizen in the evolving landscape of anatomy education. In this study, our objectives were twofold: 1) to assess the effectiveness of incorporating games in the first-year medical curriculum for reinforcing anatomy knowledge, and 2) to explore whether game-based sessions elicit improved student responses in the learning of anatomy.

Methodology: A total of 100 first-year Bachelor of Medicine and Bachelor of Surgery (MBBS) students at All India Institute of Medical Sciences (AIIMS), Bibinagar, Hyderabad, Telangana, India, were exposed to game-based learning which involved six rounds: acronym mnemonics (Redolent), jigsaw puzzle solving (Dumbfound), Filling gaps in concept maps (Blogging), Connecting images (Kinship), case scenario creation (Penman), and rapid-fire round (Rattling).

Results: At the end of the intervention, a test was taken and feedback was obtained from all the participants using a prevalidated questionnaire prepared based on a 5-point Likert scale. Questionnaire responses were subjected to descriptive analysis, and reliability analysis (Cronbach’s α) was performed to evaluate the internal consistencies of items. A paired t-test indicated that there was a significantly large difference between before (mean (M) = 17.2, standard deviation (SD) = 9.1) and after (M = 25.9, SD = 8), t(99) = 18.4, p < .001, signifying that the performance of the students was far better with game-based learning approaches than conventional learning.

Conclusion: Combining game-based education with Kaizen principles in anatomy education not only prepares students for success in their academic pursuits but also empowers them to navigate the complexities of the ever-evolving healthcare landscape with confidence and proficiency.

## Introduction

Kaizen is a Japanese philosophy of continuous improvement in the workplace. It can be applied to a wide range of scenarios and for personal growth or enhancing life skills. Kaizen in education isn't just about improvement; it's about ongoing betterment involving everyone and emphasizes the importance of continuous follow-up and teamwork. A collaborative approach strengthens communication skills, hones the ability to negotiate differences, and instills a shared responsibility for continuous improvement. The concept of Kaizen in medical education involves applying the principles of continuous improvement to enhance student development and learning outcomes. By adopting Kaizen practices for problem-solving, we can empower students to develop essential life skills and achieve sustainable academic results [1]. Educational games are structured learning activities with set rules and a competitive aspect. Recently, medical educators have shown increased interest in incorporating games into teaching. Many card and board games have been developed to teach specific topics in nursing, veterinary, and medical programs. Games adapted from TV quiz shows are also used to encourage participation in large group settings. Games, due to their interactive nature, can be powerful tools for active learning. The main goal of active learning is to engage students and prompt reflection on their work, which can be achieved through well-designed games. Decision-making, a crucial skill, is inherent in all types of games and contributes to long-term memory retention. Using games as teaching tools encourages open communication among students and enhances their ability to explain ideas and reasoning to each other [2].

Anatomy is fundamental in medicine, but it's often considered tough to understand and remember. However, if we create well-thought-out anatomy games, learning can become enjoyable and interesting for students. Unlike traditional teaching methods, game-based learning aims to make learning more engaging and interesting by incorporating game elements into non-gaming areas. Using creative and interactive methods, we can center the learning process around the students, encouraging them to actively participate, think critically, and apply their knowledge. Game-based education can significantly contribute to incorporating Kaizen methods in education by enhancing student engagement, motivation, and collaboration. The study explores the significance of continuous improvement through Kaizen in the evolving landscape of anatomy education. The objective of our study was to investigate whether game-based learning sessions, combined with Kaizen strategies, enhance student engagement and improve learning outcomes in anatomy.

## Materials and methods

Study design

A quasi-experimental study (with pre-test and post-test designs) was conducted to assess the effectiveness of game-based learning and Kaizen strategies in anatomy for first-year medical students.

Participants

The target participants of the study were first-year medical students of the 2022-23 batch in the Department of Anatomy, All India Institute of Medical Sciences (AIIMS), Bibinagar, Hyderabad, Telangana, India. The study involved a total of 100 participants (66 males and 34 females). Participation in the study was voluntary, and we ensured that all students received detailed information about the study's goals while guaranteeing the confidentiality and anonymity of their personal information.

Ethics statement

This work was approved by the Institute Ethical Committee (IEC) of our institute, AIIMS, Bibinagar, with approval number AIIMS/BBN/IEC/DEC/2021/126. The participation was completely voluntary and informed consent was taken. 

Study setting and intervention

After completing the study of the thorax and abdomen regions through lectures and dissections, all the participants underwent a test. The test consisted of 30 recall-based multiple-choice questions and two clinical scenario questions, each carrying 10 marks. The time allotted for the test was one and a half hours. The marks scored by the participants were taken as pre-test scores.

**Figure 1:**
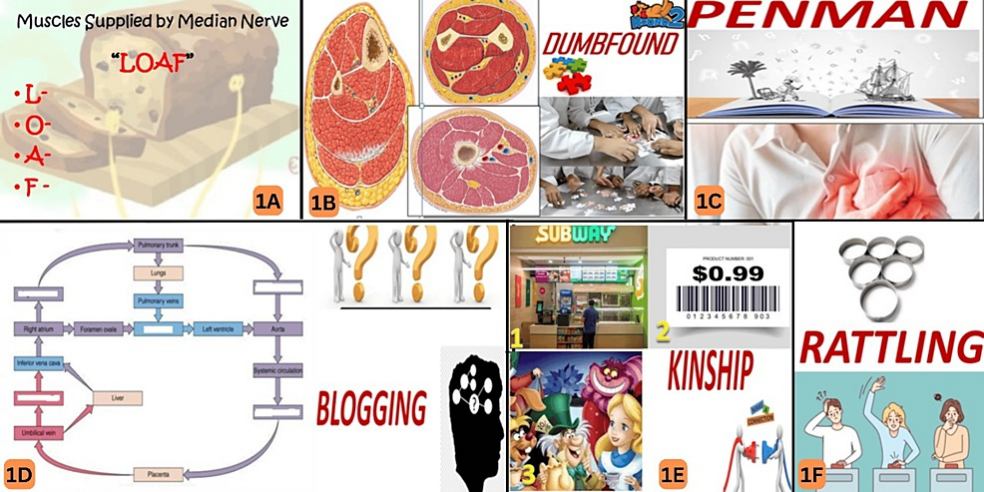
Game-based strategies for implementing Kaizen in anatomy 1A) Redolent (acronym mnemonics), 1B) Dumbfound (jigsaw puzzle solving), 1C) Penman (creating case scenario by seeing clinical images), 1D) Blogging (filling gaps in concept maps), 1E) Kinship (connexions), 1F) Rattling (rapid-fire round)

In the subsequent tutorial session, the suggested intervention plan was implemented. The participants were divided into five teams, each comprising 20 members, using a simple sampling method. The session took place in a classroom, with PowerPoint slides (Microsoft Corporation, Redmond, Washington, United States) displayed on a large screen and facilitated by the anatomy faculty. The program consisted of six rounds (Figure 1).

Round 1: Redolent (Acronym Mnemonics)

Acronyms (both word and syllabic acronym types) were projected and the participants were expected to give an answer in a stipulated time of thirty seconds (eg., Voice of America for venacaval, oesophageal, aortic openings in the diaphragm).

Round 2: Dumbfound (Jigsaw Puzzle Solving)

Each team was given a jigsaw puzzle with 30 pieces of colored atlas images of the cross-sectional anatomy of different regions and the team was given ten minutes to complete the puzzle.

Round 3: Blogging (Filling Gaps in Concept Maps) 

A series of concept maps with higher order difficulty but with limited time for each team to solve them ensured that a team succeeded only if they worked together. Each team was given a concept map with gaps, which they had to fill in a stipulated time of 30 seconds.

Round 4: Kinship (Connecting Images) 

Each team was given a set of images; they had to guess the right names for the images and link them to get anatomical terms. For example, in a given question, the first image was of a subway (food court), the next image showed the barcoding cost of one product, and the final image showed the cartoon character Alice from 'Alice in Wonderland.' Now the students would have to connect the names of these images as sub+cost+alice = subcostalis. This was a funny round where students would have to connect general vocabulary to anatomical terms and answer in 30 seconds.

Round 5: Penman (Case Scenario Creation From Images)

Images of clinical conditions like angina, inguinal hernia, varicose veins, pleural effusion, and Klumpke ’s palsy were projected, one for each team, to create case scenarios, which would give impetus to their creative ideas and align them with their clinical knowledge.

Round 6: Rattling (Rapid-Fire Round) 

Single-answer types of questions that required definitive answers (eg., what is the root value of phrenic nerve?) were included to assess the level of accuracy of the knowledge of the learner. Each team was provided with 20 questions to be answered in two minutes.

Each direct question for the team was awarded full marks if answered correctly; questions were passed once to the next team in rounds 1, 3, and 4 only, and awarded half the marks if they answered correctly. The marks were displayed on a scoreboard to motivate them and to keep the awarding system transparent. At the end of the intervention, feedback was taken from all the participants using a prevalidated questionnaire prepared based on a 5-point Likert scale.

The day after completing the intervention, all participants took an assessment that followed the same question pattern as the previous test. The marks scored by the participants were taken as post-test scores.

Data analysis

A prevalidated questionnaire for feedback comprising both closed-ended and open-ended questions prepared based on a 5-point Likert scale was used to assess the overall perceptions of the interactive game-based collaborative learning strategies of the students. The content validity of the questionnaires was evaluated through an examination by five senior academic staff with experience matching the research topic. Accordingly, the content validity index for each item was calculated, and input from the validators was considered to make the necessary amendments. The questionnaire had two sections. The first section consisted of questions about students’ overall perceptions of the interactive collaborative learning strategies and team-based activities in the quiz. In the next section, questions were asked to get the students' feedback about each round of the quiz on a Likert scale. Questionnaire responses were subjected to descriptive analysis, and reliability analysis (Cronbach’s α) was performed to evaluate the internal consistencies of items.

Quantitative and qualitative data were collected, and appropriate statistical analysis was done using GraphPad InStat version 3.1 software (GraphPad Software, La Jolla, California, United States). Mean (M) and standard deviation (SD) were used to measure the quantitative variables, and any significant differences in the outcomes were analyzed using the paired student's t-test. The M score was calculated for the close-ended statements with the Likert scale response. For the open-ended questions, content analysis was used to identify, interpret, and obtain themes for students' responses.

## Results

In the present study, 100 students participated voluntarily including 66 males and 34 females. All students were of age group 19-22 years. The results of the paired-t test indicated that there was a significantly large difference between before (M = 17.2, SD = 9.1) and after (M = 25.9, SD = 8), t(99) = 18.4, p < .001. The result signified that the performance of the students was far better with game-based learning approaches than with conventional self-learning (Table 1).

**Table 1:** Comparison of students’ performance in pre-test and post-test SD: standard deviation

Pre-test scores (n=100)		Post-test scores (n=100)		Computed t value	p-value
Mean	SD	Mean	SD	18.43	0.03783
17.18	9.06	25.9	8		

**Figure 2:**
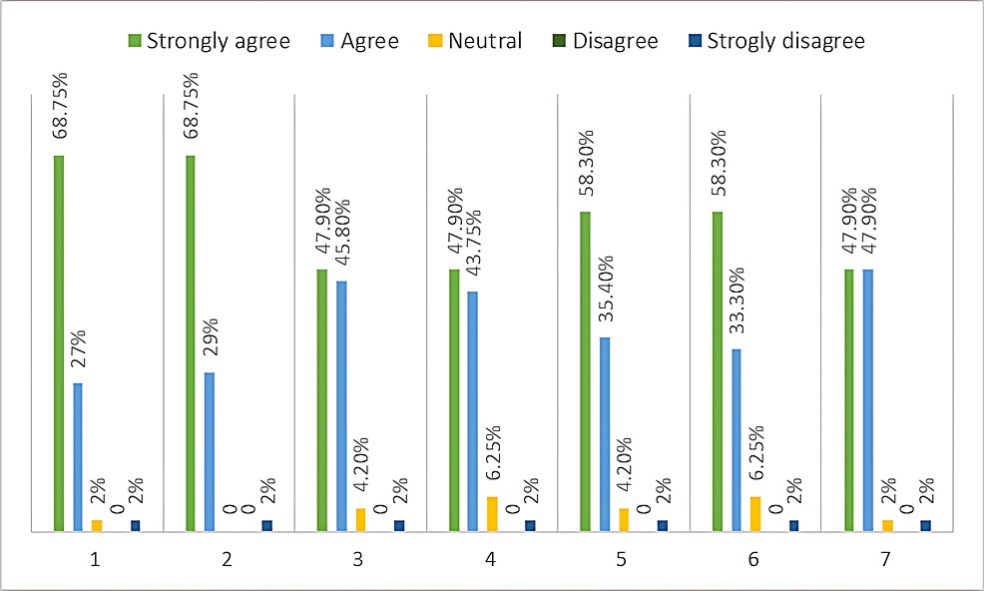
Responses of the participants to various questions asked about the whole session on a 5-point-basis Likert scale 1. Interactive game-based learning motivates me to learn more. 2. Interactive game-based learning is a fun and interesting method of learning anatomy and embryology. 3. Interactive game-based learning activities help to retain my knowledge. 4. The discussion during this session helped me improve my understanding of the subject and correct my misconceptions. 5. This session made me understand the importance of teamwork and helped develop team spirit. 6. Solving the problems in a group is an effective way to reinforce what I have learned. 7. This session helped me develop a mutual respect for other teammates' viewpoints.

Table 2 and Figure 2 present participants’ responses to items on their perceptions of game-based interactive learning strategies and the reliabilities of each sub-scale. Respondents generally agreed with statements that game-based interactive learning was useful for student learning and it helped them improve their understanding of the subject, correct the misconceptions, and retain their knowledge, where the median responses were 4. The median responses to team spirit ranged from 3.33 to 3.67. Cronbach’s α values of the instrument were >0.8 for all seven subscales, which demonstrated acceptable levels of reliability.

**Table 2:** Respondent’s perception of interactive game-based learning strategies SD: standard deviation

Subscale	Mean + SD	Median reliability	(Cronbach’s α)
Interactive game-based learning motivates me to learn more	4.67 + 0.51	5	0.86
Interactive game-based learning is a fun and interesting method of learning anatomy and embryology	4.69 + 0.46	5	0.88
Interactive game-based learning activities help to retain my knowledge	4.44 + 0.57	4	0.87
The discussion during this session helped me improve my understanding of the subject and correct my misconception	4.42 + 0.61	4	0.86
This session made me understand the importance of teamwork and helped develop team spirit	4.55 + 0.57	5	0.86
Solving the problems in a group is an effective way to reinforce what I have learned	4.53 + 0.61	5	0.88
This session helped me develop a mutual respect for other teammates' viewpoints	4.46 + 0.54	4	0.88

**Figure 3:**
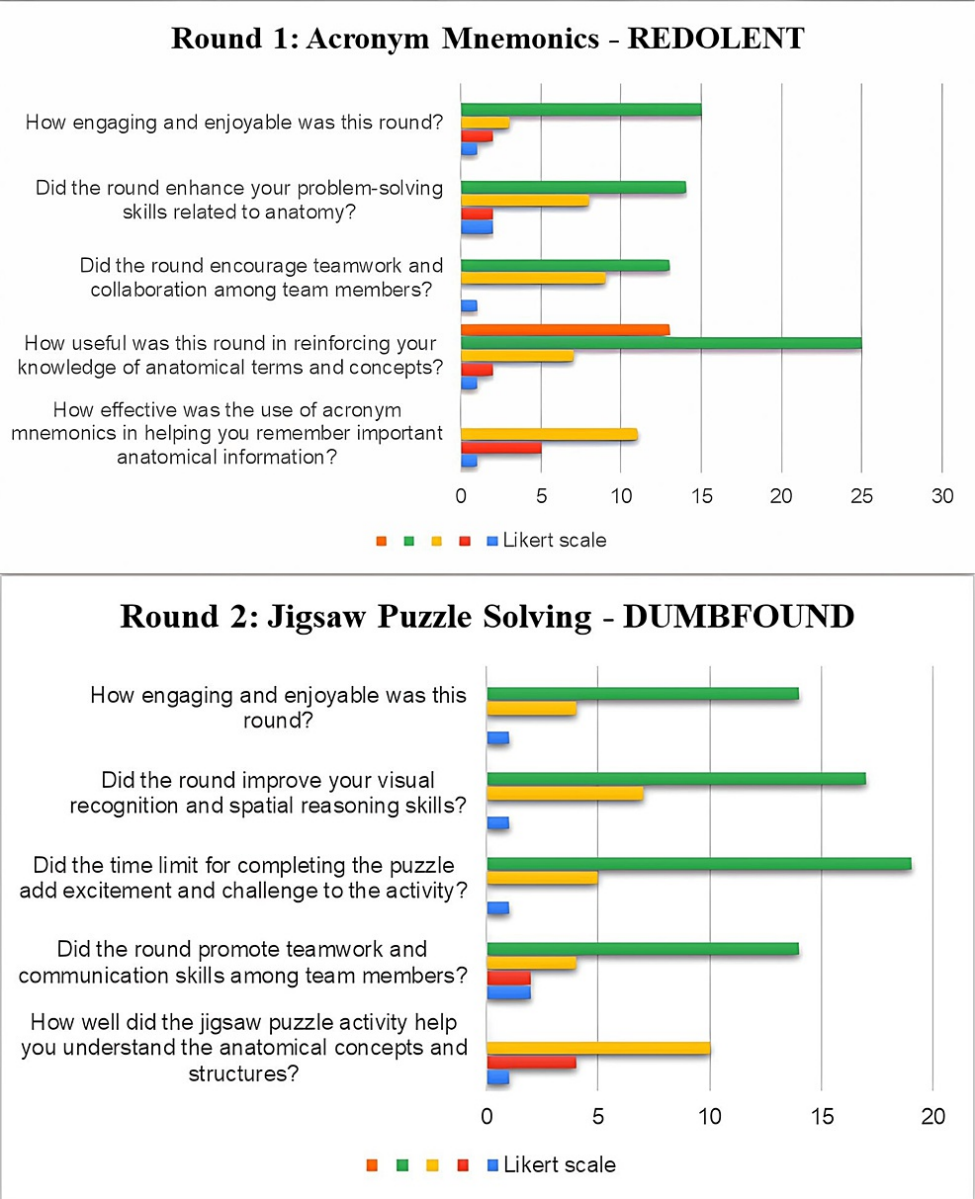
Responses of the participants in rounds 1 and 2

**Figure 4:**
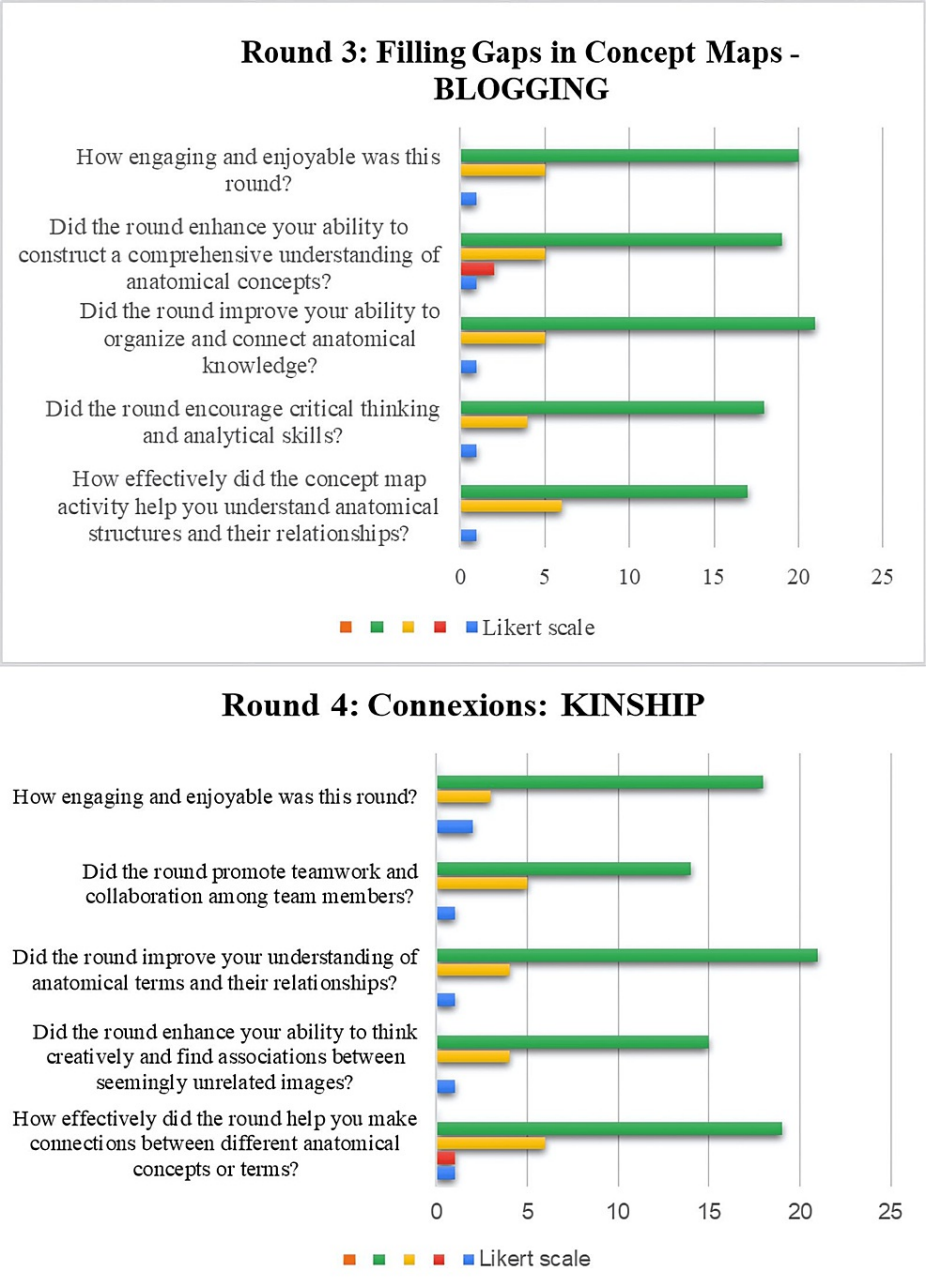
Responses of the participants in rounds 3 and 4

**Figure 5:**
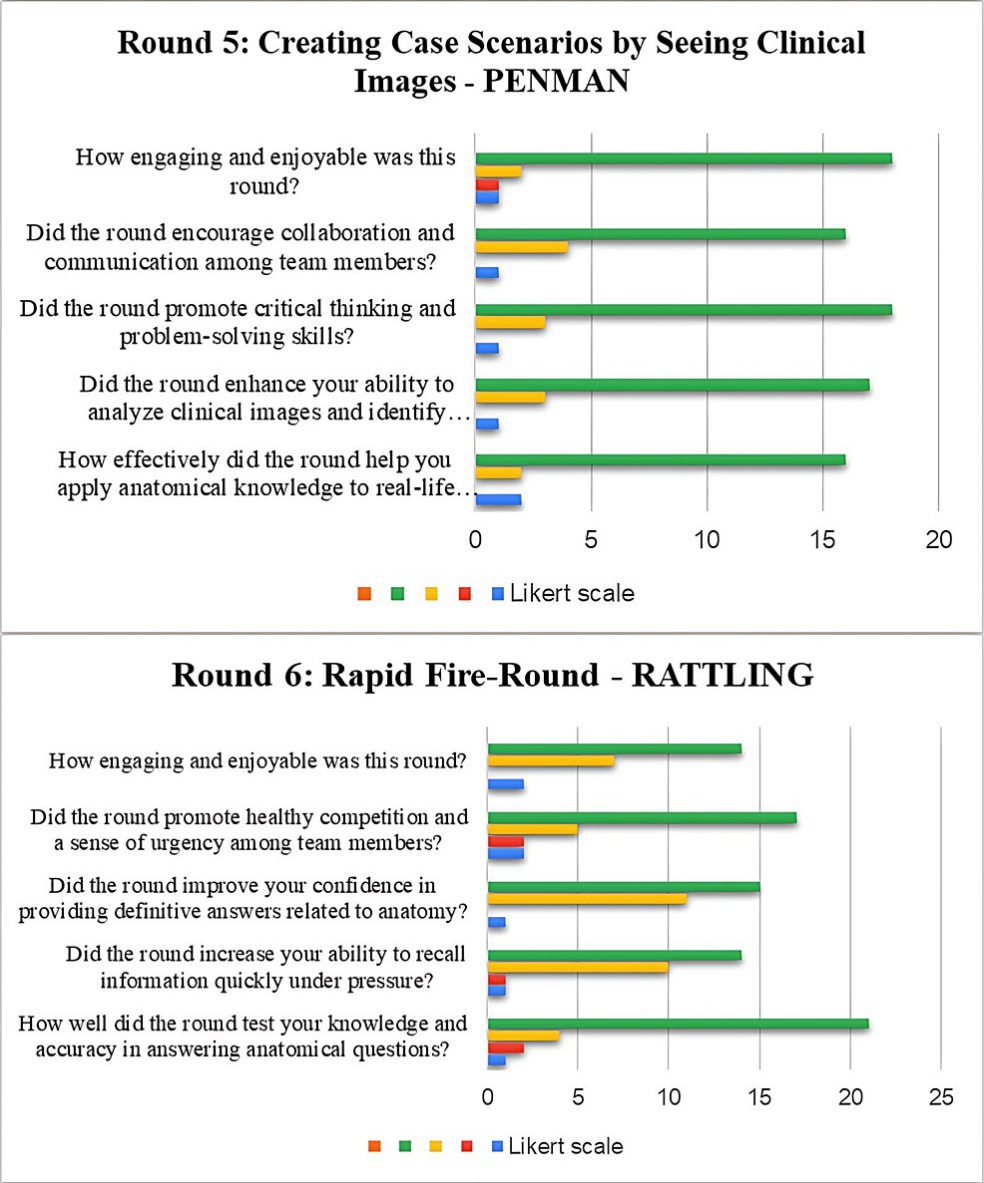
Responses of the participants in rounds 5 and 6

Responses to section two of the feedback were recorded in the graphs (Figures 3, 4, 5).

According to 33.3% of the participants, the use of the acronym mnemonics (round 1) helped them to remember important anatomical information, and 31.25% agreed that the use of mnemonics helped them to reinforce the anatomical terms and concepts. About round 2 of the quiz, 35.4% said that the jigsaw puzzle activity helped them to understand the anatomical concepts and structures very well, and 54% believed that the round promoted teamwork and communication skills among the members. Most of the participants found this round engaging and enjoyable; 48% of the students felt that the time limit for completing the puzzle added excitement and challenge to the activity, and 47.9% believed that this quiz round improved their visual recognition and spatial reasoning skills.

In round 3, half of the participants (50%) said that the concept map activity helped them to understand the anatomical structures and their relationships, 52% strongly agreed that this round encouraged them to critically think and they helped them to utilize their analytical skills. Regarding round 4, the connexion round, 43.75% strongly agreed that the round helped them to make connections between different anatomical concepts or terms, 58.33% strongly agreed that it was helpful in enhancing their ability to think creatively and find associations between seemingly unrelated images, 45.8% agreed that the round improved their understanding of the anatomical terms and their relationships, and 58.33% said that the round promoted teamwork and collaboration among the team members.

Creating case scenarios by seeing clinical images was round 5, where 58.33% strongly agreed that the round helped them to apply anatomical knowledge to real-life clinical scenarios; 56.25% believed that the round enhanced their ability to analyze clinical images and identify diagnostic conditions. In Round 6, the rapid-fire round, 43.75% of students agreed that this round helped them to test their knowledge and accuracy in answering anatomical questions, 45.8% strongly agreed that this round increased their ability to recall information quickly, 43.75% strongly agreed that the round improved their confidence in providing definitive answers related to anatomy, and 45.8% said that this round promoted healthy competition and a sense of urgency among team members.

**Figure 6:**
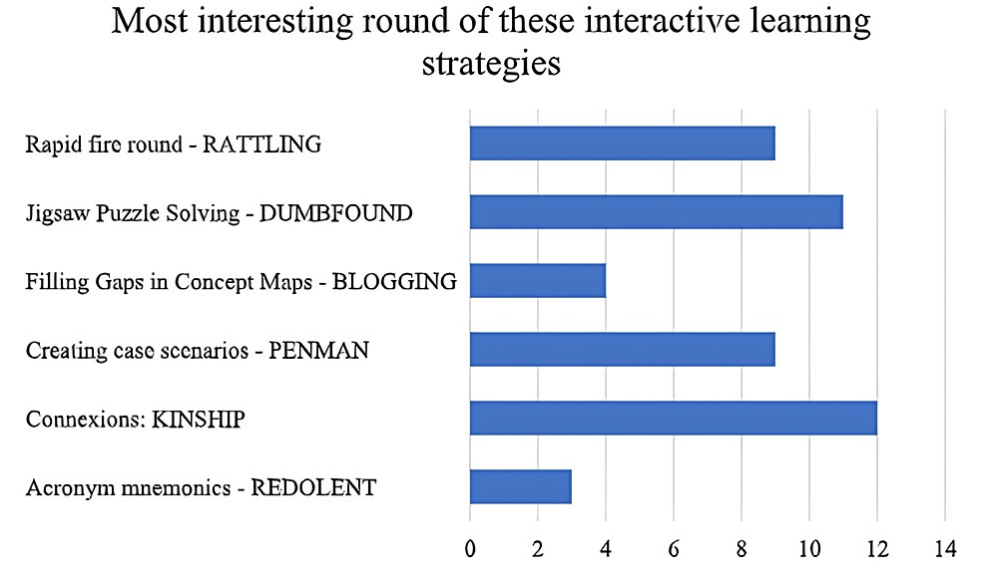
Overall response on the most interesting round out of the various interactive learning strategies used in the study.

Overall, connexions and jigsaw puzzle-solving rounds were liked by the majority of the students, followed by the rapid-fire round and the round of creating case scenarios (Figure 6).

## Discussion

In the current study, the paired t-test analysis indicated a statistically significant difference in performance, with students scoring significantly higher after the intervention. This suggests that the game-based learning approach had a positive impact on the students' understanding of anatomy and embryology compared to traditional self-learning methods. The feedback collected from students through Likert scale-based questionnaires provided valuable insights into their perceptions of the interactive collaborative learning strategies. A majority of students expressed positive views, emphasizing that the strategy motivated them to learn definitively, made learning fun and interesting, and enhanced their understanding of the subject. Additionally, students recognized the importance of teamwork and problem-solving in a group, indicating the effectiveness of collaborative learning.

Around 68.75% of the students opined that the adopted interactive strategies were fun and interesting and greatly helped them in the reinforcement and retention of the subject. The detailed analysis of each round in the feedback section revealed specific strengths of each activity. The collaborative nature of the game-based sessions promotes teamwork, communication skills, and problem-solving, which are all essential elements in the Kaizen philosophy.

Acronym mnemonics (round 1: Redolent)

The utilization of mnemonic acronyms echoes the Kaizen principle of making material unforgettable for effective retention and retrieval. Mnemonics are learning techniques that are specifically designed to make material unforgettable for effective retention and retrieval. About one-third (33%) of the participants found that mnemonics served as a memory aid, reinforcing anatomical terms and concepts. A study assessing the effectiveness of mnemonics for tertiary students found that mnemonics are useful for helping students recall information and reducing stress, aligning with the use of the acronym mnemonics in the anatomy quiz [3]. Another study demonstrated that mnemonic acronyms can improve learning and performance of procedural tasks, suggesting potential benefits for anatomy education [4]. Studies have documented that they assist students in remembering, consolidating, and learning new information [5].

Jigsaw puzzle solving (round 2: Dumbfound)

Jigsaw puzzle-solving is a constructive method to understand the underlying anatomy including important attributes of concept and form. Students have long been driven by word puzzles, hidden messages, crosswords, and word scrambles. These have been utilized to enhance logical thinking and problem-solving abilities. It increases pupils' alertness, attention, and creativity and aids in the development of their cognitive and analytical abilities [5].

A total of 54% of students opined that the activity helped them improve their visual recognition and spatial reasoning skills and facilitated them to visualize the concept image. They also expressed that the time limit provided for such activities helped them develop team spirit and promoted teamwork and communication skills among team members. A time limit calls for engagement and participation from the students. Aside from this, students have a great feeling of pride after solving the problem and take personal responsibility for their education [5].

The effectiveness of the jigsaw educational method in academic settings has been studied by many authors [6,7]. A meta-analysis explored the use of the jigsaw method in cooperative learning among health science students and concluded that it led to improvements in cognitive and non-cognitive skills [8]. A study exploring puzzle game-based learning to promote the understanding of surgical principles concluded that it helped in improving knowledge and cognitive function in students [9]. This round correlates with Kaizen by fostering logical thinking, problem-solving abilities, and teamwork. The time limit imposed during this activity resonates with the Kaizen emphasis on engagement and participation, contributing to a sense of pride and personal responsibility for learning.

Filling up gaps in concept maps (round 3: Blogging)

This was an implied method of reinforcing knowledge gained regarding anatomical structures and their relations, connections, and constructivism of the concept. Concept maps offer a distinctive viewpoint to learning since they use diagrams to illustrate a range of concepts and have precise links with one another [10].

Approximately 52% of students expressed that the concept maps helped them understand anatomical structures and their relationships better, helped them organize and connect the anatomical knowledge, and enhanced their ability to construct a comprehensive understanding of anatomical concepts. They found it effective in seeing the big picture and chunking information based on meaningful connections while reinforcing the understanding of anatomical terms. This round encouraged critical thinking and analytical skills. A study found that it was an effective tool for long-term memorization; it enhanced students' mastery and recollection of class material, providing insights into the effectiveness of concept maps in improving learning outcomes in anatomy among first-year general medicine students [11]. The students opined that these concepts made them realize the importance of anatomical learning with coherency. They felt that the application of concept maps in learning modality on a regular basis would help them retain the subject longer. Studies have also documented that the mapping approach is effective, particularly when it comes to long-term memory in the study of anatomy [12]. However, limited time can hinder the ability to construct a comprehensive understanding of complex relationships in concept maps for a few students. This round aligns with Kaizen's focus on reinforcing knowledge and creating connections. The collaborative effort in constructing a comprehensive understanding reflects the principles of continuous improvement. Students' recognition of the effectiveness of concept maps in long-term memorization resonates with Kaizen's aim of sustained learning outcomes.

Connexions (round 4: Kinship)

Enhanced creative thinking helped in finding associations between seemingly unrelated images, promoting teamwork and collaboration. The use of visual aids, teamwork, and creative thinking in round 4 aligned with the principles of effective collaborative learning strategies. Collaborative learning strategies, such as the jigsaw technique, think-pair-share, and peer review, have been shown to enhance student engagement, promote teamwork, and improve knowledge retention [13,14]. The Kinship round supports Kaizen by enhancing creative thinking and encouraging associations between seemingly unrelated images. The emphasis on teamwork and collaboration in this round reinforces the Kaizen principle of collective responsibility for improvement. This also provides an effective device for quicker memorization, linking complex anatomical terms and concepts with familiar cartoon and movie characters. However, the disagreement of a few participants could be due to the unfamiliarity with the cartoon and movie characters or difficulty in making connections between medical and non-medical terms.

Creating case scenarios (round 5: Penman)

Over half of the participants strongly agreed that this round helped them apply anatomical knowledge to real-life clinical scenarios and analyze clinical images. The activity enhanced their ability to think critically and apply theoretical knowledge to practical clinical situations [15,16]. Similar studies in the literature have also shown positive results in enhancing medical students' learning experiences and clinical skills when case scenarios and clinical images were used. For example, a study by Plackett et al., found that students in their study significantly scored higher in the clinical image-based case scenario group in terms of learning outcomes [17]. This round resonates with Kaizen by emphasizing the application of anatomical knowledge to real-life clinical scenarios. This activity promotes critical thinking and practical application, aligning with Kaizen's holistic approach to improvement.

Rapid-fire round (round 6: Rattling)

A rapid-fire quiz is an innovative, interactive teaching and learning tool that uses a question-and-answer structure to promote healthy competition and active involvement [18]. In the present study, around 43.75% of the participants opined that this round promoted a sense of healthy competition among them and the time limit provided to answer the questions instilled a sense of urgency among the team members. More than half of the participants felt that the round was challenging, testing their accuracy in understanding anatomy. However, around 40% of the participants felt that it was quite difficult to recall their knowledge when they were under pressure as time constraint was imposed upon them in answering the questions. This reflects the Kaizen principle of promoting healthy competition and active involvement. While the time constraint may pose a challenge, it aligns with the Kaizen philosophy of instilling a sense of urgency among team members, fostering continuous improvement in knowledge recall and accuracy.

Limitations

Limited time for each round in the session may have affected students' performances and perceptions. Variability in prior knowledge, learning styles, and external factors such as stress were not controlled for. So, it could have influenced the results.

## Conclusions

Anatomy education is a crucial component of medical education, and it is essential to ensure that students have a positive learning experience. By combining game-based education with Kaizen principles in anatomy education, educators can create a dynamic learning environment that not only enhances students' understanding of anatomy but also equips them with essential life skills such as adaptability, creativity, and resilience. This approach not only prepares students for success in their academic pursuits but also empowers them to navigate the complexities of the ever-evolving healthcare landscape with confidence and proficiency. The use of various teaching methods, such as mnemonic acronyms, jigsaw puzzle-solving, concept maps, connections, case scenarios, and rapid-fire rounds, have been found to be effective in reinforcing anatomical knowledge and improving various skills. However, challenges still exist in anatomy education, such as difficulty in memorizing content and a lack of opportunity to apply knowledge. It is important to explore the perceptions and views of anatomy educators and other stakeholders to improve anatomy teaching and learning. Further research is needed to comprehensively reveal the views of education stakeholders and to examine the effectiveness of different teaching methods. Overall, anatomy education is a dynamic field that requires ongoing evaluation and improvement to ensure that students receive the best possible education.
